# A Transfer Learning-Based Framework for Classifying Lymph Node Metastasis in Prostate Cancer Patients

**DOI:** 10.3390/biomedicines12102345

**Published:** 2024-10-15

**Authors:** Suryadipto Sarkar, Teresa Wu, Matthew Harwood, Alvin C. Silva

**Affiliations:** 1 Department Artificial Intelligence in Biomedical Engineering, Friedrich-Alexander-Universität Erlangen-Nürnberg, 91054 Erlangen, Germany; 2 School of Computing and Augmented Intelligence, Arizona State University, Tempe, AZ 85281, USA; teresa.wu@asu.edu; 3 Mayo Clinic, Department of Radiology, Phoenix, AZ 85259, USA; harwoodml@gmail.com (M.H.); silva.alvin@mayo.edu (A.C.S.)

**Keywords:** prostate cancer, lymph node metastasis, deep learning, magnetic resonance imaging, machine learning

## Abstract

**Background:** Prostate cancer is the second most common new cancer diagnosis in the United States. It is usually slow-growing, and when it is low-grade and confined to the prostate gland, it can be treated either conservatively (through active surveillance) or with surgery. However, if the cancer has spread beyond the prostate, such as to the lymph nodes, then that indicates a more aggressive cancer, and surgery may not be adequate. **Methods:** The challenge is that it is often difficult for radiologists reading prostate-specific imaging such as magnetic resonance images (MRIs) to differentiate malignant lymph nodes from non-malignant ones. An emerging field is the development of artificial intelligence (AI) models, including machine learning and deep learning, for medical imaging to assist in diagnostic tasks. Earlier research focused on implementing texture algorithms to extract imaging features used in classification models. More recently, researchers began studying the use of deep learning for both stand-alone feature extraction and end-to-end classification tasks. In order to tackle the challenges inherent in small datasets, this study was designed as a scalable hybrid framework utilizing pre-trained ResNet-18, a deep learning model, to extract features that were subsequently fed into a machine learning classifier to automatically identify malignant lymph nodes in patients with prostate cancer. For comparison, two texture algorithms were implemented, namely the gray-level co-occurrence matrix (GLCM) and Gabor. **Results:** Using an institutional prostate lymph node dataset (42 positives, 84 negatives), the proposed framework achieved an accuracy of 76.19%, a sensitivity of 79.76%, and a specificity of 69.05%. Using GLCM features, the classification achieved an accuracy of 61.90%, a sensitivity of 74.07%, and a specificity of 42.86%. Using Gabor features, the classification achieved an accuracy of 65.08%, a sensitivity of 73.47%, and a specificity of 52.50%. **Conclusions:** Our results demonstrate that a hybrid approach, i.e., using a pre-trainined deep learning model for feature extraction, followed by a machine learning classifier, is a viable solution. This hybrid approach is especially useful in medical-imaging-based applications with small datasets.

## 1. Introduction

According to the American Cancer Society, prostate cancer is the second most common cancer in American men after skin cancer. As per their estimates, about 248,530 new cases and 34,130 deaths occurred from prostate cancer in 2021 alone [1]. Often, prostate cancer is slow-growing and initially confined to the prostate gland [2]. In these instances, patients may need no treatment, opting instead for active surveillance. Other patients may need surgery, chemotherapy, immunotherapy, radiation therapy, or often a combination of these. The decision to intervene, and the best intervention, hinges on the cancer’s stage. Staging cancer depends on primary tumor growth, for example, growth into adjacent organs such as the seminal vesicles or the urinary bladder. Staging also depends on the secondary, metastatic extent. Prostate cancer has a predilection for spreading to pelvic/retroperitoneal lymph nodes and bones. As the overall prostate cancer tumor burden often determines the treatments offered, reliable staging is important, and medical imaging often plays a key role [3,4]. Magnetic resonance imaging (MRI) has emerged as an important imaging modality for the assessment of tumor invasion and pelvic lymph node metastases [5]. However, the determination of lymph node metastatic status can be challenging because abnormal (cancerous) and normal lymph nodes often appear similar on MRI. As such, the sensitivity of imaging for lymph node metastasis in prostate cancer is low [6].

To address the aforementioned challenges, artificial intelligence (AI)-based research has gained special prominence in medical imaging diagnosis. Earlier efforts mainly focused on texture analysis as a means of feature extraction from medical images, which were in turn fed into machine learning models for classification. Texture analysis refers to the segregation of the different regions in an image, based on their physical characteristics or intensity distribution. There are different texture analysis algorithms, including but not limited to the gray-level co-occurrence matrix (GLCM), local binary patterns (LBPs), and Gabor filters. Researchers often utilize texture-based algorithms in combination with features that are representative of the imaging modality in question [7]. For example, ref. [8] designed a multifractal feature descriptor to classifying non-neoplastic tissues and tumors, as well as to grade hepatocellular carcinoma tissues into five stages. For both of those tasks, the proposed feature descriptor outperformed GLCM-, LBP-, and Gabor-based features. Ref. [9] utilized features from the GLCM, LBP, and Gabor filters to classify benign vs. malignant pulmonary nodules using the Support Vector Machine (SVM) classifier. The results showed a similar performance accuracy of 90.00% for all three of these features but a highest area under the curve (AUC) of 92.70% for GLCM. Ref. [10] made use of a dataset of 22 patients with glioblastoma to perform classification between true progression and pseudoprogression from T2-weighted MRI images. Five GLCM features, namely homogeneity, entropy, energy, correlation, and contrast were extracted, out of which correlation generated the best classification performance, with an accuracy of 86.40%, an AUC of 89.20%, an sensitivity of 75.00%, and a specificity of 100%. Ref. [11] reported a sensitivity of 98.00%, a specificity of 99.25%, an accuracy of 99.07%, and an AUC of 99.80% using GLCM and morphological features classified by the k-Nearest Neighbor–Cosine (KNN-Cosine) classifier, for the detection of carcinoma on prostate cancer patients. The GLCM features extracted were contrast, correlation, dissimilarity, energy, entropy, homogeneity, mean, variance, standard deviation, skewness, kurtosis, and root mean squared (RMS)—each extracted along four different orientations ($0^{\circ }$, $45^{\circ }$, $90^{\circ }$, and $135^{\circ }$). The morphological features extracted include area, perimeter, maximum radius, minimum radius, Euler number, eccentricity, equivalent diameter, elongatedness, entropy, circularity 1, circularity 2, compactness, dispersion, standard deviation, and shape index. Ref. [12] made use of GLCM-based features (namely contrast, homogeneity, difference variance, dissimilarity, and inverse difference) for predicting the Gleason score (GS) of patients with prostate cancer from T2-weighted standard MRI images. Three GS groupings were defined based on range: G1 when GS≤6; G2 when GS=3+4; and G3 when GS≥4+3. The authors reported a prediction AUC of 78.40% for G1, 82.35% for G2, and 64.76% for G3. Ref. [13] made use of contrast and homogeneity GLCM features, in combination with mean, median, and 10th- and 25th-percentile values, to predict prostate cancer aggressiveness from T2-weighted (T2w) and Apparent Diffusion Coefficient (ADC) MRI scans of 45 patients before prostatectomy. Out of these 45 patients, 41 had one clinically significant tumor focus, and 4 had two clinically significant tumor foci. The study reported AUCs of 94.50% and 96.20%, for T2w and ADC, respectively. Ref. [14] utilized Gabor and LBP features to perform Gleason grading of 160 prostate cancer patients from H&E stained histological images into four Gleason categories, namely benign, grade 3, grade 4 and grade 5. The study reported a performance accuracy of 98.30% with the 10-fold KNN classifier. Ref. [15] made use of texture analysis for the detection of prostate cancer from MRI and Magnetic Resonance Spectroscopy (MRS) scans. Haar features were extracted from the MRS images, and Gabor features were extracted from the MRI scans. Subsequently, dimensionality reduction was performed, followed by classification using the Random Forest (RF) classifier, which provided an AUC of (89.00 ± 2.00)%. Ref. [16] utilized texture features to perform prostate cancer detection on digital biopsies. A total of 10 features were finally chosen using an AdaBoost ensemble method, from an initially extracted set of 900 first-order statistical, second-order statistical, and Gabor features, for the purpose of prostate cancer detection on 100 images extracted from 58 patients. The study reported AUCs of 84.00%, 83.00%, and 76.00% on low, medium, and high image resolutions, respectively. Ref. [17] performed feature extraction using first-order statistical features, second-order statistical, or co-occurrence matrix features and Gabor filter-based features and achieved a best sensitivity of 78% and a False Positive Rate (FPR) of 6% with the combined feature set for prostate cancer diagnosis.

While texture analysis-based investigation is still underway, it is worth noting that recent efforts in deep neural-network-based approaches have gained popularity among researchers owing to their automatic feature extraction capabilities from images. The most common type of deep neural network is the convolutional neural network (CNN), which has been used extensively in imaging, video and audio applications. CNNs have been studied for detection, classification and segmentation tasks in medical research. For example, ref. [18] utilized three different CNN architectures (Inception V3, Inception-ResNet V2 and ResNet-101) to detect axillary lymph node metastasis from primary breast cancer patients. Inception V3, the best-performing model, reported an area under curve (AUC) of 89.00%, a sensitivity of 85.00%, and a specificity of 73.00%. In comparison, the radiologists achieved 73.00% sensitivity and 63.00% specificity. Ref. [19] made use of eight different pre-trained CNN models to diagnose cervical lymph node metastasis from 995 axial CT scans of patients with thyroid cancer. Res-Net-50 was the best-performing model, with an AUC of 95.30% and an accuracy of 90.40%. Ref. [20] utilized ten CNN-based architectures on a small dataset of 218 patients to perform classification on lymph node metastasis. They reported a mean AUC of 68.00%, an accuracy of 61.00%, a sensitivity of 53.00%, and a specificity of 70.00%. The research reviewed above employed end-to-end neural-network-based approaches wherein training and validation were performed on the same dataset. A prevalent criticism against such approaches is that they require large datasets to achieve desirable performance. Researchers therefore increasingly employ the concept of transfer learning—that is, pre-training a deep model on a large imaging dataset (such as ImageNet), and subsequently fine-tuning the weights on smaller target data. Table 1 provides a summary of the proposed work, in comparison to similar works from the literature.

In this research, since the dataset is small (126 lymph node images), instead of taking an end-to-end deep learning approach, we decided to make use of transfer learning to address issues relating to overfitting. We utilized a CNN model, ResNet-18, which was pre-trained on ImageNet (a public dataset with a large volume of data) and then transferred to our prostate image dataset to extract features. The features were then utilized to perform classification between normal and metastatic lymph nodes. For the purpose of dimensionality reduction on our relatively small dataset, we developed a feature selection procedure comprising supervised and unsupervised feature selection techniques. The most important features obtained from the feature selection step were subsequently used for classification using the 10-fold decision tree (DT) classifier, which achieved an accuracy of 76.19%, a sensitivity of 79.76%, a specificity of 69.05%, a precision of 83.75%, and an F1-score of 81.71%. In addition, to investigate the efficacy of the features extracted from the pre-trained deep model over texture features, we implemented two texture analysis algorithms: GLCM and Gabor. Following the same workflow in terms of feature selection and classification, the DT classifier trained on GLCM features achieved its best classification accuracy of 61.90%, a sensitivity of 74.07%, a specificity of 42.86%, a precision of 71.43%, and an F1-score of 72.73%. The same DT, when trained on Gabor features, achieved a best classification accuracy of 65.08%, a sensitivity of 73.49%, a specificity of 52.50%, a precision of 76.25%, and an F1-score of 74.84%. These sets of experiments show that the proposed combined deep-learning-machine-learning architecture is promising for the automatic classification of normal vs. metastatic prostate cancer lymph nodes.

## 2. Materials and Methods

First, we performed feature extraction from the image regions of interest (ROIs) using a pre-trained ResNet-18 deep neural network (we also extracted texture-based features for the purpose of comparison). Next, we performed feature selection, which was particularly important because our dataset contains very few samples and is therefore prone to overfitting without proper feature selection. Subsequently, we performed classification between cancer and normal ROIs using statistical machine learning models.

**Figure 1:**
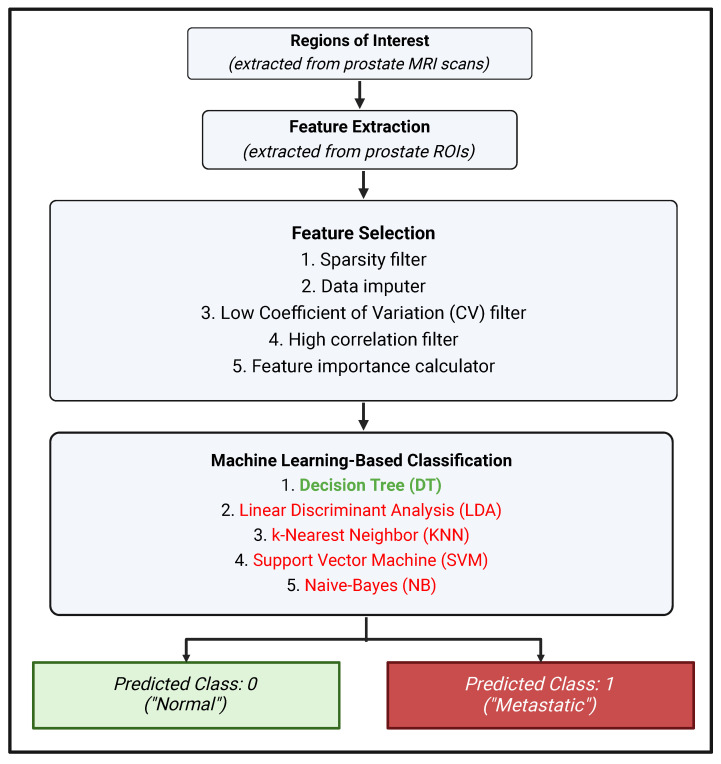
A schematic representation of the overall workflow: feature extraction, feature selection, and classification. It is to be noted that although five classifiers were initially used to differentiate “metastatic” and “normal” lymph nodes, only the decision tree classifier was retained for the final analyses because all of the other classifiers had sub-par performance. This makes sense because the feature selection framework utilized Random Forest, which is a boosted decision tree algorithm.

Figure 1 provides a schematic representation of the overall workflow, starting from the raw images of lymph nodes to their automatic categorization as normal vs. metastatic. The methodology includes feature extraction, feature selection, and classification. In the proposed hybrid framework, we first extracted a 512-element feature vector per image from the average pooling layer of the ResNet-18 pre-trained model. For the purpose of comparison, we also extracted features using two texture analysis-based approaches, namely GLCM and Gabor. Next, we implemented our feature-selection algorithm on this feature vector. The decision tree (DT) classifier was finally trained on the selected features.

### 2.1. Feature Extraction

#### 2.1.1. Texture Feature Extraction

Two texture algorithms for feature extraction were implemented: GLCM and Gabor. For GLCM, 11 features, namely contrast, correlation, energy, entropy, homogeneity, variance, sum of average, sum of variance, sum of entropy, difference of variance, and difference of entropy, were extracted from each of the ROI scans. The scalar distance was selected as s = 2, and the orientations were selected as $\theta =0^{\circ },45^{\circ },90^{\circ }$, and $135^{\circ }$ corresponding to 4 different GLCM matrices. Subsequently, the corresponding features from each orientation were averaged to generate the final 11 GLCM features. For Gabor, the feature extraction methodology was inspired by [22]—where a filter bank of 40 filters (five different scales, with eight orientations per scale) was utilized to extract features. A uniform input image size of 28 × 28 was used, resulting in a total of 28 × 28 × 40 = 31,360 features extracted per ROI scan. Subsequently—in order to remove the redundancy of features extracted [22,23,24]—we decided to downsample the features by a scale of four, thereby reducing the feature vector size to 31,360/(4 × 4) = 1960 features per scan.

#### 2.1.2. ResNet-18 for Feature Extraction

**Figure 2:**
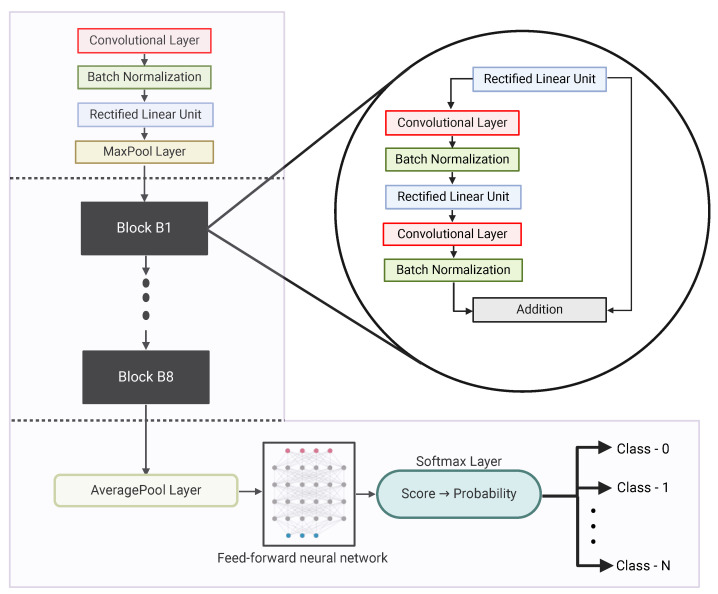
ResNet18 model: it comprises a total of 71 layers, and the trained weights from the “average pooling” layers are used for classification.

A 71-layer ResNet-18 model pre-trained on the ImageNet dataset was used to extract features from the raw scans. Weights from the fifth and last pooling layer were extracted and used as weights for classification between malignant and non-malignant scans. Note that the weights obtained using this approach were concatenated to generate a 512-element feature vector corresponding to each lymph node scan. Figure 2 provides a pictorial representation of the ResNet-18 model that has been utilized in this work. The model comprised a total of 71 layers, out of which the trained weights from the “average pooling” layer were used for classification.

### 2.2. Feature Selection Mechanism

We employed an ensemble technique to select the most important features from the originally extracted feature vector—which was obtained using the pre-trained ResNet-18 model, GLCM, and Gabor filter banks, respectively, to perform the classification of normal vs. metastatic lymph nodes. The steps involved in the feature selection process are described in detail in Table 2. We employed the Random Forest algorithm, one of the most widely used algorithms in machine learning for the purpose of classification as well as feature selection.

### 2.3. Machine Learning Classification

The selected features obtained from the feature selection algorithm were fed into a machine learning classifier to differentiate between normal vs. metastatic lymph nodes. In order to investigate the classification performance of the proposed model, 10-fold cross-validation (CV) was utilized. The dataset was randomly shuffled and split evenly into 10 different groups. In each iteration, nine of these groups were used for training, and the remaining one was used for testing. The iteration process was repeated 10 times, and each time, the test set was a different group. The results obtained across the 10 iterations were finally averaged and reported. It must be noted that the groups were generated in such a manner so as to ensure that the training and test sets were mutually exclusive of one another (that is, no overlap of samples). The decision tree (DT) classifier was the only classifier that performed well in differentiating metastatic versus normal lymph nodes on the selected feature set, which makes sense because the feature selection itself was performed by Random Forest, which is a boosted decision tree algorithm.

The decision tree is a hierarchical, non-parametric, supervised learning algorithm that learns via a series of if–then–else decisions. Each node in a decision tree represents a “test” (also known as “question”) on a particular **feature**, which in the case of a binary decision tree, can be visualized as a coin toss. The edge entering a particular node represents the decision made in the previous step, and the edges leaving the node represent the “decisions” (formally known as “outcomes”) being made at the current node (in the case of a coin toss test, the two outcomes are *heads* and *tails*). Each node in a decision tree, via training, learns the optimal threshold of the **feature** associated with that particular node, which maximizes classification performance.

The training set contains a set of characteristics (also known as “features”—the same as the *features* described above)—and corresponding labels—often depicted as $\left[X,y\right]=(x_{1},x_{2},x_{3},\ldots ,x_{F},$$\ldots x_{n-1},x_{n})\in R^{m\times (n+1)}$. These particular training data comprise a total of *n* features, $x_{F}$ depicting feature *F*, and a label associated with each training sample—the label set as a whole depicted as $y\in R^{m\times 1}$; the training set here has a total of *m* samples.

There are several techniques for computing impurity metrics that help decide the optimal split at each node, the two most commonly used being Gini impurity and Shannon’s entropy. In this work, we utilized the Gini index.

Machine learning classifiers try to improve performance by reducing impurity, in other words, maximizing information gain. Information gain is formally defined as follows:

$$IG_{X,X_{F}}\left(X,f\right)=I\left(X\right)-I\left(X/f\right)$$

where, $IG_{X,X_{F}}\left(X,f\right)$ is the information gain (also known as mutual information) of a random variable (r.v.) *X* resulting from an r.v. $X_{F}$ assuming the value of *f*; I(X) is the impurity measure of r.v. *X* obtained in the previous step, i.e., before r.v. $X_{F}$ assuming the value of *f*; I(X/f) is the conditional impurity measure of r.v. *X* given that the value of r.v. $X_{F}=f$.

Here, r.v. *X* denotes the set of all features associated with the training set defined above, and *f* everywhere in the equation denotes feature $X_{F}\;inX$ assuming the value of *f*.

The decision tree algorithm tries to learn the optimal value of feature $X_{F}$ by learning $X_{F}=f$ such that I(X/f) is minimized, thereby maximizing information gain $IG_{X,X_{F}}\left(X,f\right)$.

### 2.4. Evaluation Metrics

In order to evaluate the efficacy of the classification model, the following five metrics were used:

(1) $$Accuracy=\frac{TP+TN}{TP+TN+FP+FN}$$

(2) $$Sensitivity=\frac{TP}{TP+FN}$$

(3) $$Specificity=\frac{TN}{TN+FP}$$

(4) $$Precision=\frac{TP}{TP+FP}$$

(5) $$F1=\frac{2\times Precision\times Sensitivity}{Precision+Sensitivity}$$

where TP is the # of true positives, TN is the # of true negatives, FP is the # of false positives, and FN is the # of false negatives. In addition, the area under the curve (AUC) was used. For detailed formulations on AUC, interested readers are referred to [25].

## 3. Software and Tools

**MATLAB R2021b** was used in order to extract features and subsequently perform machine-learning-based classification of the imaging data. (Five pre-trained models, namely AlexNet, GoogleNet, InceptionV3, ResNet50, and XceptionNet, all which are available as part of the Deep Learning Toolbox of Matlab, were used for neural-network-based classification. The built-in Statistics and Machine Learning Toolbox of Matlab was used to train four classifiers, namely decision tree, Linear Discriminant Analysis, Support Vector Machine, and Naive Bayes.) The feature selection framework and code for plot generation were programmed using **Python** (version 3.7). Data cleaning, pre-processing, and a majority of the image processing framework was developed using the same installation of Python. **ImageJ** (version 1.53) was used to visualize and analyze the grayscale images. **BioRender** [26] was used for the purpose of figure generation for the paper.

## 4. Dataset

**Figure 3:**
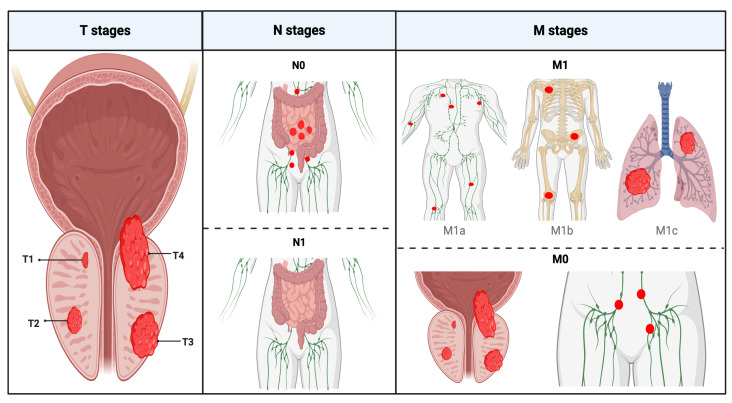
A pictorial explanation of the TNM cancer staging protocol as it pertains to prostate cancer.

A radiological dataset was provided by Mayo Clinic, Scottsdale, Arizona. The dataset comprised multiple de-identified gray-level MRI scans of lymph nodes, obtained using a varied range of diffusion-weighted image (DWI) contrast types from a prospective clinical trial of 15 high-risk (Gleason ≥ 8) prostate cancer patients. The patients underwent prostate MRI before prostatectomy and pelvic lymph node dissection as part of a trial. The tissue was submitted for pathologist review. The location of each lymph node was confirmed, and labels of the pre-operative MRI data were generated. The labels were “metastatic” (meaning harboring metastatic cancer cells) and “normal” (meaning no cancer metastases were found). As per the TNM cancer staging protocol (see Table 3 and Figure 3), for this particular project, we only focused on M1 (labeled “metastatic”) versus M0 (labeled “normal”) classification.

**Figure 4:**
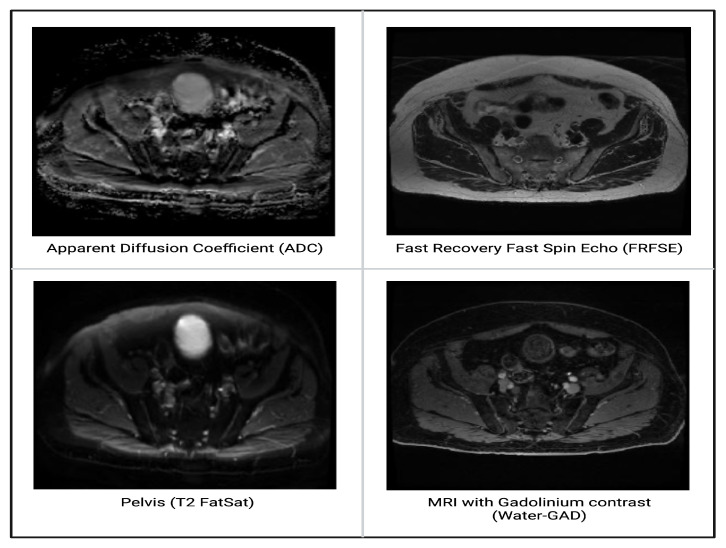
A prostate MRI from the same patient; four MRI sequences are shown.

There were a total of 126 lymph node images: 41 metastatic and 85 normal. The four MRI sequences, each with different tissue contrast characteristics, were as follows: Apparent Diffusion Coefficient (ADC), Fast Recovery Fast Spin Echo (FRFSE), Pelvis (T2 FatSat) and MRI with Gadolinium Contrast (Water-GAD). Figure 4 snlws a prostate MRI from the same patient; four MRI sequences are shown. Table 4 provides an overview of the lymph node scans, distributed over the four aforementioned MRI sequences (ADC, FRFSE, Pelvis, Water-GAD) and two class labels (metastatic, normal).

**Figure 5:**
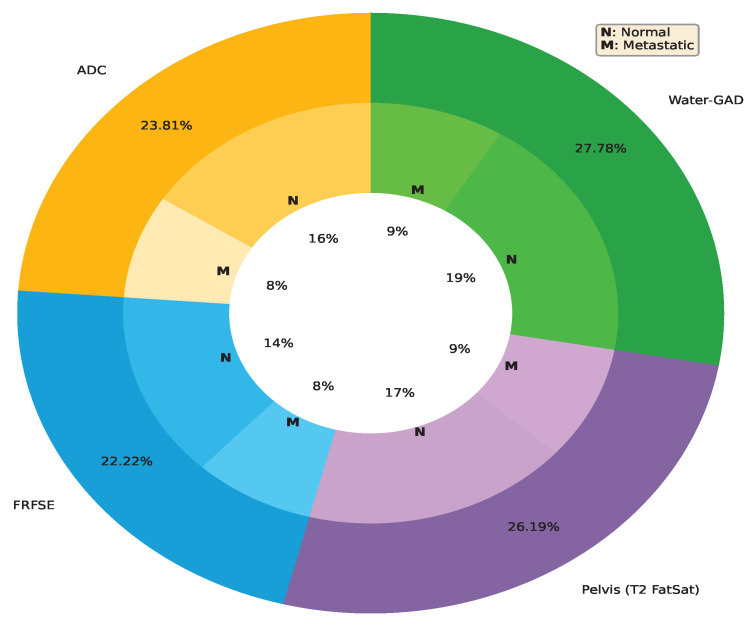
The distribution of normal and metastatic samples in each of the four MRI sequences, namely ADC, FRFSE, Water-GAD and Pelvis (T2 FatSat). The percentages in the inner circle represent the number of samples under each label–sequence combination as a percentage of the total number of samples present in the entire dataset, whereas the percentages in the outer circle represent the distribution of images across the four MRI sequences.

The dataset comprises normal and metastatic samples at a ratio of approximately 2:1 (85 normal, 41 metastatic). The distribution of normal and metastatic samples in each of the four the MRI sequences is consistent with the overall data (see Figure 5).

### 4.1. Signal Weighting

An isotropic DWI can be obtained from an ADC image and an image with signal intensity of b=0 using the following formulation:

(6) $$S_{b}=S_{0}e^{-bD}$$

where $S_{b}$ is the diffusion-weighted image, $S_{0}$ is the image with no diffusion weighting (b=0), *b* is the level of diffusion weighting that has been applied to the image signal, and *D* is the ADC image, also known as the diffusion coefficient signal.

#### 4.1.1. ADC

ADC voxel values (also known as the *diffusion coefficient*) are calculated from a series of conventional DWI images and are meant to show a greater level of diffusion than DWIs by getting rid of the T2-weighting coefficient.

As can be derived from Equation (6), an ADC image can be obtained from an isotropic DWI image and an image with signal intensity of b=0 using the following formulation:

(7) $$D=-\frac{1}{b}log_{e}\left(\frac{S_{b}}{S_{0}}\right)$$

where the symbols $S_{b}$, $S_{0}$, *b* and *D* are the same as defined in Equation (6).

#### 4.1.2. FRFSE

FRFSE is a T2-weighted MRI contrast type that has consistently and significantly managed to reduce imaging time while maintaining T2 weighting since the Fast Spin Echo (FSE) and Turbo Spin Echo (TSE) techniques developed in the 1990s, both of which are T2W imaging techniques as well [27]. There are two main types of FRFSE image acquisition techniques: the breath-hold (BH) technique; and the respiratory triggering (RT) technique—also known as the non-breath-hold technique. The BH-FRFSE technique is especially popular in imaging and identifying liver lesions [27,28]. It also helps reduce image artifacts, owing to having a short imaging time [29]. Several studies have shown significantly better performances of BH techniques in comparison to RT techniques in general [30] and BH-FRFSE in particular when compared against primitive non-BH techniques such as the RT-FSE [29]. BH-FSE-based techniques have been shown to cut down on image acquisition time, reduce artifacts, improve lesion sizing, and capture better structural characteristics as compared to their traditional, Spin Echo (SE) counterparts.

#### 4.1.3. T2 FatSat

The T2w Fat Saturation (T2 FatSat) technique helps detect a minimal presence of water in the pelvis. Fats often mimic high-signal-intensity fluids, thereby making it difficult to distinguish the two. The T2 FatSat protocol makes distinguishing fats from pelvic fluids, particularly those with high signal intensities, easier [31].

#### 4.1.4. Water-GAD

Water-GAD is a T1-weighted MRI contrast type, imaged using Gadolinium metal as a contrast agent. T1w images bring out the differences in T1 relaxation times between different types of tissue. Fat shows up in the form of bright voxels, while water shows up in the form of dark voxels, in T1w images.

### 4.2. Image Preprocessing

Min–max normalization was performed on each image individually, in order to account for varying intensity ranges across the database. Additionally, gradient-descent-based optimization algorithms have not only been shown to converge significantly faster [32] on scaled data compared to unscaled data, but also perform better [33,34]. Since our proposed classification model extracts data using pre-trained neural networks, stochastic gradient descent is extensively used. Although we do not expect to see a conceivable difference in runtime complexity, owing to the small sample size of our data when scaled on bigger datasets, the difference is expected to be significant. Moreover, feature scaling has also been shown to aid in improving the speed, performance, and often both during the process of convergence in various statistical machine learning algorithms [33,34], for example, Linear Discriminant Analysis [35], kNN [33,36,37], SVM [38], and Naive Bayes [39]. In fact, decision trees (and their variants such as the Random Forest classifier, which is a boosted decision tree algorithm) are among the very few mainstream statistical learning-based algorithms that are completely independent of the variance in the training data and therefore fare no better in terms of performance or optimization time whatsoever with feature scaling [33].

Min–max normalization transforms features to fall in the range of [0,1], in this context, meaning the lowest pixel value in the transformed image is 0 and the highest pixel value is 1.

Each pixel *p* in the normalized image matrix $X^{\prime }$ (denoted as $X_{p}^{\prime }$), can be obtained from the corresponding pixel *p* in the original image matrix *X* (denoted as $X_{p}$) using the following formulation:

(8) $$X_{p}^{\prime }=\frac{X_{p}-min\left(X\right)}{max(X)-min(X)}\forall p\in \left[1,n_{p}\right]$$

where $n_{p}$ is the total number of pixels in the original (and normalized) image, min(X) is the value of the pixel with the lowest intensity in the original image *X*, and max(X) is the value of the pixel with the highest intensity in the original image *X*.

We performed min–max normalization on each image in the dataset using scikit-learn’s sklearn.preprocessing.MinMaxScaler function, with the default parameter settings: feature_range=(0, 1), copy=True, and clip=False.

## 5. Results

Using the pre-trained ResNet-18 model, 512 features were originally extracted, 9 of which were selected using our aforementioned feature selection mechanism. For GLCM, 11 features were originally extracted, out of which 5 features were finally selected. For Gabor, 1960 features were first extracted, 15 of which were ultimately selected. Table 5 summarizes the classification performance of the DT classifier on the original and selected features from ResNet-18, GLCM, and Gabor. Using the selected features, features selected from the ResNet-18 features outperform both GLCM and Gabor. Specifically, using DT as a classifier, (1) Resnet-18 reported the highest classification accuracy of 76.19%, a sensitivity of 79.76%, a specificity of 69.05%, a precision of 83.75%, and an F1-score of 81.71%; (2) GLCM achieved an accuracy of 61.90%, a sensitivity of 74.07%, a specificity of 42.86%, a precision of 71.43%, and an F1-score of 72.73%; (3) Gabor achieved an accuracy of 65.08%, a sensitivity of 73.49%, a specificity of 52.50%, a precision of 76.25%, and an F1-score of 74.84%. In addition, feature selection helped significantly improve performance compared to classification on original feature sets. It was observed that (1) for ResNet-18, the improvements in accuracy, sensitivity, specificity, precision, and F1-score were 19.05%, 17.41%, 22.71%, 13.08%, and 15.46%, respectively; (2) for GLCM, the improvements in accuracy, sensitivity, specificity, precision, and F1-score were 3.96%, 1.23%, 9.53%, 3.61%, and 2.49%, respectively; (3) for Gabor wavelet-based features, the improvements in accuracy, sensitivity, specificity, precision, and F1-score were 5.56%, 6.02%, 5.00%, 3.52%, and 4.84%, respectively.

Table 5 provides the AUC values of each of the three sets of features before and after feature selection using the 10-fold DT classifier. The area under the curve (AUC) of the six experiments further exhibit the efficacy of the feature selection algorithm over using the original feature set for classification. Specifically, (1) the 512 original ResNet-18-based features, which provided unstable and varying AUC values across implementations, after feature selection achieved a 94.59% on the top 9 features; (2) GLCM shows a slight improvement from an AUC of 92.11% on the 11 original features to an AUC of 95.12% on the 5 selected features; (3) Gabor feature performances remain comparable between the original feature set of 1960 features, which had an AUC of 99.20%, and the selected feature set of 15 features, which had an AUC of 98.98%.

## 6. Discussion and Conclusions

The results presented in this research show that the features extracted by the pre-trained ResNet-18 model outperform both the GLCM and the Gabor filter features after feature selection. Although it is not entirely clear to us why the original ResNet-18-based features, without feature selection, achieved unstable and variable AUC values across implementations, we contend that since we have only 126 samples and 512 original features (a feature-to-sample ratio of 4:1), the model might be overfitting the training set and struggling to attain generalizability on the validation set. This problem, however, is countered by utilizing the feature selection mechanism, which, when applied to the ResNet-18 model achieves, the highest AUC of 94.59% and an F1-score of 81.71% on the top nine deep features after classification using the 10-fold DT classifier. It is also observed that all three feature extractors yield a higher accuracy, sensitivity, specificity, precision, and F1-score after feature selection than on the original set of features—thereby exhibiting the efficacy of the feature selection algorithm in general and the Random Forest feature importance calculator in particular.

The AUC of 94.59% obtained by our approach had a higher value compared to those reported in some of the prior studies relying on imaging alone. For example, [6] states that the AUC varies between 69.00% and 81.00% for prostate cancer detection over multi-parametric MRIs, which includes diffusion-weighted imaging (DWI). The authors of [40] designed an automatic deep CNN-based architecture to detect prostate cancer on diffusion-weighted magnetic resonance imaging (DWI). The database comprised DWI images of 427 patients (175 prostate cancer and 252 healthy patients). The model yielded an AUC of 87.00%. The authors of [41], the runners up of the 2017 PROSTATEx challenge, designed a deep learning model called the XmasNet, which was based on deep CNNs, for the purpose of performing classification on prostate cancer lesions utilizing 3D multiparametric MRIs. They achieved an AUC of 84.00%. The database comprised 341 patients, each having Diffusion Weighted Images (DWI), Apparent Diffusion Coefficient (ADC) scans, Ktrans, and T2w images. The authors of [42] designed a radiomics signature by selecting 9 out of 150 manually extracted GLCM and gray-level histogram features from the lymph node CT scans of 118 patients. A radiomic nomogram was subsequently generated using the logistic regression model, which achieved an AUC of 89.86%. The authors of [43] utilized a total of 103 T2-weighted MRIs to perform the classification of lymph node metastasis on bladder cancer patients. A total of 718 features—comprising textural features, shape-based features, wavelet features, and first-order statistics features—were manually extracted from the bladder scans, and a nomogram was generated using logistic regression. Feature selection was performed, which reduced the number of important features from 718 to 9. Classification was subsequently performed on these 9 important features, generating a validation AUC of 84.47%. The sensitivity of 79.76%, a specificity of 69.05%, accuracy of 76.19%, and an AUC of 94.59% obtained by our approach performed significantly better than the sensitivity of 53.00%, the specificity of 70.00%, theaccuracy of 61.00%, and the mean AUC of 68.00% reported in [20], which utilized ten CNN-based architectures on a small dataset of 218 patients to perform the classification of lymph node metastasis.

In medical imaging research, one of the primary challenges is that interpretability varies greatly between radiologists. The PI-RADS architecture is pervasively utilized for the purpose of image interpretation. However, ref. [44] exhibits many impending issues of inter-observer interpretability associated with the PI-RADS model. Owing to the innate difficulties associated with identifying anomalies in prostate MRIs, there seems to be varying consensus among researchers and radiologists alike, with respect to determining the best identification methodologies. Sample size—as in the case of our study as well—often proves to be an important factor that determines the selection of the classification model. While we know that machine learning performs better on datasets with limited samples, we also acknowledge the capability of deep models to extract more meaningful features. In [45], the authors demonstrated that on a dataset of multiparametric MRIs obtained from 52 prostate cancer patients, hierarchical clustering performed better than deep models in differentiating between normal and tumor prostate tissues.

While the statistical measures that we have employed to evaluate our model seem to work well, we acknowledge that there might be more medically sound metrics for measuring performance, which, to a certain degree, predict the chances of survival as well. In order to evaluate such parameters, we need to have an in-depth understanding of associated biomedical processes, as well as access to a wide range of radiological features. The authors of [46] have identified certain measures and classifiers that outperform others when it comes to prostate cancer with lymph node metastasis. A sample size of 1400 patients—all with metastatic prostate cancer of the lymph node—was employed in this study. Univariate analysis revealed that age, Gleason score, radiotherapy history, T stage, log odds of metastatic lymph node (LODDS) classifier, lymph node ratio (LNR) classifier, and number of metastatic lymph node (NMLN) classifier except for the total number of lymph nodes examined (TNLE) were some of the most consequential predictors of patient survival. Multivariate analysis suggested that LODDS, LNR, and NMLN except the TNLE classifiers were some of the most important parameters for measuring survival rate.

## 7. Future Work

As with most medical imaging data, our dataset also suffered from a significant class-wise imbalance of samples. The negative class contained 84 ROIs, which was twice that of the positive class (42 ROIs). Therefore, the training process was heavily biased in favor of the negative class. The problem of imbalanced classes was addressed by employing the Synthetic Minority Oversampling Technique (SMOTE), which is used to generate synthetic instances of the class with fewer samples. However, in this case, the artificially generated samples further confused the training model, thereby yielding even poorer results. Simple physical transformations of the ROIs such as rotation, flipping, et cetera were also applied to increase the sample size of the normal class but without any improvements in classification performance. In the future, we intend to extend this work by performing neural-network-based ROI augmentation, which includes the application of generative adversarial networks (GANs).

In addition to performing the classification of lymph node metastasis in prostate cancer patients, we also intend to extend our analysis to subsequently be able to localize the lesion region in lymph node images where cancer has been detected. From previous experience working on similar medical imaging datasets, we have noticed that having an additional set of “difference features” helps in not only localizing the lesion region but also monitoring the progression of lesion over time. This involves subtracting time-variant images from fixed baseline data. In this case, since we have a control image of an unaffected portion of the lymph node provided, corresponding to every cancerous region of the lymph node, we could use the control as a baseline image to then perform localization. However, for that to work effectively, we need more positively labeled (or cancerous) samples. Currently, we have too few lesion lymph node images to train a full-fledged model to perform localization. We are in the process of acquiring more samples to enable us to explore the use of the classifier in cascade with the localization framework, which is a future effort.

## Figures and Tables

**Table 1:** A comparison between the proposed work and similar works from the literature. Unless specified in the *Database* column, all data used in the referenced literature have been acquired by the authors themselves.

Literature	Task	Database	Performance
Iqbal et al. [11]	Classification of carcinoma vs. healthy tissue	Diffusion-weighted MRIs, brachytherapy images	Accuracy: 99.07% AUC: 99.80% Sensitivity: 98.00% Specificity: 99.25%
Chaddad et al. [12]	Prediction of GS	Diffusion-weighted MRIs (obtained from [21])	AUC (G1): 78.40% AUC (G2): 82.35% AUC (G3): 64.76%
Vignati et al. [13]	Prediction of prostate cancer aggressiveness	Diffusion-weighted MRIs	AUC (T2w): 94.50% AUC (ADC): 96.20%
Farooq et al. [14]	Automatic GS grading	H&E stained biopsy tissue.	Accuracy: 98.30%
Tiwari et al. [15]	Classification of prostate cancer patients vs. healthy patients	T2w MRIs, MRSs	AUC: (89.00±2.00)%
Doyle et al. [16]	Classification of cancer vs. healthy tissue micro- environments in prostate cancer patients	H&E stained biopsy tissue	AUC (high resolution scans): 84.00% AUC (medium resolution scans): 83.00% AUC (low resolution scans): 76.00%
Nguyen et al. [17]	Classification of prostate cancer patients vs. healthy patients	H&E stained biopsy tissue	Sensitivity: 78.00% FPR: 6.00%
Wessels et al. [20]	Classification of lymph node meta- stasis in primary prostate cancer patients	H&E stained images, pathology data	Accuracy: 61.00% AUC: 68.00% Sensitivity: 53.00% Specificity: 70.00%
Proposed work	Classification of lymph node meta- stasis in prostate cancer patients	Diffusion-weighted MRIs	Accuracy (ResNet-18 features): 76.19% AUC (ResNet-18 features): 94.59% Sensitivity (ResNet-18 features): 79.76% Specificity (ResNet-18 features): 69.05% Precision (ResNet-18 features): 83.75% F1-score: 81.71% Baseline comparison performances with GLCM and Gabor features available in the Results (and briefly, in the Introduction) section of the paper.

**Table 2:** Steps involved in the feature selection process.

***Step 1. Missing Value Filter:*** First, we need to remove any missing values that might be present in the feature matrix. Through a procedure of trial and error, we determined that we should drop all features having at least 20% of the values missing (in this case, zero).
***Step 2. Imputation:*** We impute missing values of the remaining features by the average of the respective feature.
***Step 3. Low coefficient of variation (CV) filter:*** This particular step is based on the idea that features with higher variance have more information contained within them. The coefficient of variation is the standard deviation normalized by the mean. This metric is very effective in taking care of the characteristic differences in the range of values among the features, thereby minimizing any bias that might have arisen from the utilization of raw feature values. The coefficient of variation is the standard deviation normalized by the mean, formally defined as $CV=\frac{\sigma }{\mu }$ where σ is the standard deviation and μ is the mean of the respective column (here, the feature weight from the pre-trained neural network).
***Step 4. High correlation filter:*** This step drops columns with a correlation value greater than 95%. This is achieved by calculating the cross-correlation matrix of all of the feature pairs and subsequently dropping one of the features with a pairwise correlation value greater than 0.95 (the feature that has a higher correlation with the dependent variable, in this case, the metastatic/ normal labels that have been provided along with the data).
***Step 5. Feature importance calculator:*** This is the most important step in the feature selection process. We made use of machine learning to mine out the features that are most consequential in forecasting the output variable (in this case, the metastatic/normal labels). First, a sklearn.ensemble.RandomForestRegressor model was trained with 1000 decision trees, and feature importances were extracted using *scikit-learn*’s sklearn.ensemble.RandomForestRegressor.feature_importances_ function. Next, *scikit-learn*’s sklearn.inspection.permutation_importance function was used to compute and rank the feature importances of the same previously mentioned RandomForestRegressor model that had been trained on 1000 decision trees. The results using both of the feature importance estimators were exactly the same, as expected. And the top ten features from this step, which contained more than 90% of the information as the original weights, were used for classification in the subsequent step.

**Table 3:** Explanation of the tumor (T), node (N), and metastasis (M) stages.

Tumor (T)	Node (N)	Metastasis (M)
**T1**: Undetectable via scans or physical examinations; sometimes detected via biopsies.	**N0**: Tumor is confined to the prostate gland; however surrounding lymph nodes do not contain any malignant tissue.	**M0**: Tumor is restricted to the pelvic region.
**T2**: Detectable via scans, physical examinations and biopsies; completely inside prostate gland.	**N1**: Lymph nodes in the immediate vicinity of the prostate gland contain malignant tissue.	**M1a**: Tumor present in lymph nodes in non-pelvic regions of the body.
**T3**: Spread to seminal vesicles surrounding the prostate.		**M1b**: Tumor present in the bones.
**T4**: Nearby organs exhibit malignant tissue, such as the bladder.		**M1c**: Tumor present in organs outside of the pelvic region (very commonly the lungs; very rarely and discovered post mortem the brain or the heart).

**Table 4:** An overview of the lymph node scans, distributed over the aforementioned four MRI sequences (ADC, FRFSE, Pelvis, Water-GAD) and two class labels (metastatic, normal).

Patient ID	ADC		FRFSE		Pelvis (T2 FatSat)		Water-GAD	
	Normal	Metastasis	Normal	Metastasis	Normal	Metastasis	Normal	Metastasis
2663	4	3	4	3	4	3	4	3
2664	2	0	1	0	2	0	2	0
2665	0	0	0	0	2	1	2	1
2666	1	0	0	0	1	0	1	0
2667	1	0	1	0	1	0	1	0
2668	1	0	2	0	2	0	2	0
2669	1	7	1	7	1	7	1	7
2670	1	0	1	0	1	0	1	0
2673	2	0	2	0	2	0	2	0
2674	1	0	1	0	1	0	1	0
2677	2	0	0	0	1	0	2	0
2678	2	0	2	0	1	0	2	0
2679	1	0	1	0	1	0	1	0
2681	0	0	1	0	1	0	1	0
2685	1	0	1	0	1	0	1	0

**Table 5:** The performance of the 10-fold DT classifier on the features (original vs. selected) extracted using ResNet18, GLCM, and the Gabor wavelet filter.

Classifier	Accuracy (%)	Sensitivity (%)	Specificity (%)	Precision (%)	F1-Score (%)	AUC (%)
ResNet-18 (original)	57.14	62.35	46.34	70.67	66.25	—
ResNet-18 (selected)	76.19	79.76	69.05	83.75	81.71	94.59
GLCM (original)	57.94	72.84	33.33	67.82	70.24	92.11
GLCM (selected)	61.90	74.07	42.86	71.43	72.73	95.12
Gabor (original)	59.52	67.47	47.50	72.73	70.00	99.20
Gabor (selected)	65.08	73.49	52.50	76.25	74.84	98.98

## Data Availability

Data will be made available under reasonable request.
